# Phenotypic and genetic resistance to Septoria blotch disease in European wheat varieties

**DOI:** 10.1002/tpg2.70237

**Published:** 2026-03-30

**Authors:** Conor Copeland, Julio Isidro y Sánchez, Humberto Fanelli, Fiona M. Doohan

**Affiliations:** ^1^ UCD School of Biology and Environmental Science and Earth Institute College of Science University College Dublin Dublin Ireland; ^2^ Centro de Biotecnología y Genómica de Plantas (CBGP, UPM‐INIA) Universidad Politécnica de Madrid (UPM) ‐ Instituto Nacional de Investigación y Tecnologia Agraria y Alimentaria (INIA) Campus de Montegancedo‐UPM Pozuelo de Alarcón Spain

## Abstract

*Zymoseptoria tritici*, the causal agent of Septoria tritici blotch (STB), poses a critical threat to wheat (*Triticum aestivum* L.) production in temperate Europe, underscoring the need for durable genetic resistance. Here, we screened 151 winter wheat varieties at the seedling stage using a mixed inoculum of five virulent isolates. Disease severity was quantified at 14, 21, and 28 days post‐inoculation to calculate the area under the disease progress curve. We observed broad phenotypic variation, with higher susceptibility in Southern European germplasm. A genome‐wide association study identified three significantly resistant loci: IWB5774 on chromosome 2D, and IWB72742 and IWB11406 on chromosome 1B. Functional annotation mapped these markers to high‐priority candidate genes, including a TaBx3D‐like protein, the endomembrane trafficking regulator *WDR91*, and *NAP1*, a nucleosome assembly factor involved in stress signaling. These findings provide robust molecular markers for breeding and nominate specific targets for functional validation of STB resistance mechanisms.

## Core Ideas

- We screened 151 European winter wheat varieties at the seedling stage with a five‐isolate *Zymoseptoria tritici* mixture and observed wide variation in the Septoria tritici blotch (STB) response.
- Genome‐wide association analysis identified three reproducible loci associated with STB: IWB5774 (Chr 2D), IWB72742 (Chr 1B), and IWB11406 (Chr 1B), across models and seasons.
- These single‐nucleotide polymorphisms (SNPs) reside within defense‐relevant genes: a TaBx3D‐like benzoxazinoid biosynthetic gene (TraesCS2D03G0669200), WDR91 (TraesCS1B03G0499000; endomembrane trafficking), and NAP1 (TraesCS1B03G1027100; chromatin/chaperone).
- Further investigation into these genes could provide additional sources of basal genetic STB resistance.
- Prioritizing benzoxazinoid metabolism, vesicle trafficking, and chromatin regulation offers complementary routes to enhance durable, broad‐based resistance.

## Plain Language Summary

Septoria tritici blotch is a fungal disease that severely impacts wheat yields, posing a risk to farm profitability and food security. To identify wheat varieties with natural resistance, we evaluated a diverse collection of wheat lines and analyzed their DNA to locate genetic “hotspots” for defense. This genome‐wide scan identified specific DNA regions associated with significantly lower disease levels. These regions contain genes likely involved in the plant's immune response and provide practical genetic markers that breeders can use to develop stronger, more resilient wheat varieties.

**Abbreviations**
AUDPC: area under the disease progress curve
DLA: diseased leaf area
dpi: days post‐inoculation; EU, European Union;
GWAS: genome‐wide association study
LD: linkage disequilibrium
QTL: quantitative trait locus
SNP: single‐nucleotide polymorphism
STB: Septoria tritici blotch
UTR: untranslated region

## 1 INTRODUCTION

*Zymoseptoria tritici* (teleomorph *Mycosphaerella graminicola*), the causal agent of Septoria tritici blotch (STB), is a leading constraint on wheat (*Triticum aestivum* L.) production, typically reducing European yields by 5%–10% (Fones & Gurr, 2015; Savary et al., 2019). Its polycyclic nature, driven by wind‐ and splash‐dispersed ascospores, facilitates rapid spread within and between fields. Infection occurs via stomata, followed by a latent non‐necrotrophic phase that transitions to necrotrophy around 11 days post‐inoculation (dpi), producing chlorotic–necrotic lesions that reduce leaf photosynthesis and crop yield (Brennan et al., 2019; Fones & Gurr, 2015). Control relies heavily on fungicides; ∼70% of European Union (EU) fungicide inputs target STB, yet resistance to several modes of action has evolved. Regulatory constraints plus climate change are likely to intensify disease pressure and limit chemical efficacy (Cheval et al., 2017; Prahl et al., 2023; Talas et al., 2024; Torriani et al., 2015; Wieczorek et al., 2015). These trends elevate genetic resistance as the most sustainable route to durable management.

To date, 23 major genes across 14 chromosomes confer qualitative resistance to STB (Brown et al., 2015; Langlands‐Perry et al., 2022; Yang et al., 2018), but their durability is often compromised, as illustrated by erosion of effectiveness for the widely deployed *Stb16q* (Kildea et al., 2020; Morais et al., 2019). In parallel, many quantitative trait loci (QTLs) contribute partial, typically broader‐spectrum resistance and tend to be more durable than single major genes, motivating breeding strategies that pyramid qualitative and quantitative sources (e.g., Riaz et al., 2020; Tamburic‐Ilincic & Rosa, 2019; Zakieh et al., 2023).

Genome‐wide association studies (GWASs) leverage phenotypic variation across diverse panels and mixed‐model frameworks to map single‐nucleotide polymorphisms (SNPs) associated with complex traits, capturing both coding and regulatory contributors. In wheat, GWAS has resolved loci for resistance to STB, and other fungal diseases, underscoring its utility for dissecting polygenic defense (Alemu et al., 2021; Kokhmetova et al., 2025; Malosetti et al., 2021; Mikaberidze et al., 2024; Muqaddasi et al., 2019; Odilbekov et al., 2019; Ouaja et al., 2025; Singh et al., 2023; Tessmann et al., 2019; Zakieh et al., 2023). Here, we extend this work by screening STB resistance in 151 European winter wheat varieties at the seedling stage with a five‐isolate *Z. tritici* mixture and applying GWAS to identify loci and candidate genes associated with reduced disease, providing targets for functional validation and markers for breeding.

## 2 MATERIALS AND METHODS

### 2.1 Plant material

We selected 151 winter wheat (*Triticum aestivum* L.) varieties from the EU InnoVar project panel (www.h2020innovar.eu) to capture genomic diversity and geographic representation across agro‐climatic zones (AGZs) (Table S1; Figure S1): Continental (21 varieties), Maritime North (54), Maritime South (52), Mediterranean (12), and Pannonian (12) (Figures S1 and S2). Seeds were synchronized by vernalization at 4°C for 8 weeks under a 16:8‐h light:dark cycle. Vernalized seedlings were transferred to 3 L pots filled with Westland John Innes No. 2 compost (Norton's Peat), placed on raised benches in a glasshouse, and grown to heading. Heads were bagged at emergence. Spikes were harvested at maturity, threshed, and grain stored at 4°C for subsequent assays.

**Core Ideas**

- We screened 151 European winter wheat varieties at the seedling stage with a five‐isolate *Zymoseptoria tritici* mixture and observed wide variation in the Septoria tritici blotch (STB) response.
- Genome‐wide association analysis identified three reproducible loci associated with STB: IWB5774 (Chr 2D), IWB72742 (Chr 1B), and IWB11406 (Chr 1B), across models and seasons.
- These single‐nucleotide polymorphisms (SNPs) reside within defense‐relevant genes: a TaBx3D‐like benzoxazinoid biosynthetic gene (TraesCS2D03G0669200), WDR91 (TraesCS1B03G0499000; endomembrane trafficking), and NAP1 (TraesCS1B03G1027100; chromatin/chaperone).
- Further investigation into these genes could provide additional sources of basal genetic STB resistance.
- Prioritizing benzoxazinoid metabolism, vesicle trafficking, and chromatin regulation offers complementary routes to enhance durable, broad‐based resistance.

### 2.2 Fungal material

We used five Dutch isolates of *Z. tritici*, including reference isolate IPO323 (Goodwin et al., 2011; Palma‐Guerrero et al., 2017), and four virulent strains (IPO88004, IPO89011, IPO94269, and IPO90012) (Ajaz et al., 2021; Chartrain et al., 2009; Qutb et al., 2024). One‐centimeter diameter plugs from 7‐day‐old *Z. tritici* cultures were placed onto 94‐mm diameter Petri dishes filled with potato dextrose agar (PDA; HiMedia) and incubated in the dark at 20°C for 7 days. Conidia were harvested by flooding plates with 3 mL sterile water and scraping with a sterile spreader. Spore concentration was measured using a Bright‐Line hemacytometer (Sigma‐Aldrich) and adjusted to 10^6^ spores mL^−1^ with sterile distilled water. Equal volumes of the five isolates were combined in 1 L Duran bottles, and 0.02% Tween20 (v v^−1^) was added as a surfactant.

### 2.3 STB experiment

We conducted three polytunnel trials at UCD Rosemount Environmental Research Station spanning 2021 and 2022. Specifically, trials were conducted in Summer 2021, Spring 2022, and Summer 2022. Seeds were pregerminated on moistened Whatman No. 1 filter paper (Whatman International Ltd.) in 94‐mm diameter Petri dishes first at 4°C for 2 days and thereafter at 20°C for 2 days. Germinated seeds were sown in 3‐L pots filled with Westland John Innes No. 2 Potting‐On compost (Norton Peats), with four seedlings per pot. At growth stage 14 (Zadoks et al., 1974), the fourth leaf of plants was marked and sprayed with 10 mL of either 10^6^ spores mL^−1^ *Z. tritici* suspension or a 0.02% v v^−1^ Tween20 solution using a 50‐mL hand‐held mist sprayer bottle. Eight plants (two pots) were treated with the pathogen, while four plants (one pot) received the control treatment. Pots were then arranged into three randomized blocks. STB disease severity was assessed by visually estimating the percentage of the treated leaf area covered by lesions at 14, 21, and 28 days post‐treatment, as previously assessed in other studies (Ajaz et al., 2021; Fones & Gurr, 2015; Palma‐Guerrero et al., 2017). Note the other method of assessing STB—percentage leaf area bearing pycnidia at 28 dpi—was not assessed as in trial 1 it was observed that for several varieties the leaves had completely senesced at 28 dpi prior to pycnidial development (hence this assessment was not used nor completed in trials 2 and 3).

### 2.4 Trait derivation and statistical analysis

We summarized disease progression as the area under the disease progress curve (AUDPC) using the trapezoidal method: ∑[(*x_i_* + *x_i_* − 1)/2](*t_i_* − *t_i_* − 1), where *x_i_* is the disease score at timepoint *t_i_* (Wilcoxson et al., 1975). We also derived a composite resistance index by z‐scaling 28 dpi diseased leaf area (DLA) and AUDPC and averaging the scaled values. Analyses were performed in R version 4.2.1 (R Core Team, 2021). As normality could not be achieved by transformation (Shapiro–Wilk), we tested varietal differences in AUDPC using Kruskal–Wallis followed by Dunn's test with Bonferroni correction.

### 2.5 Genotyping and quality control

Accessions were genotyped using the Illumina 90K SNP array at the North Central Small Grains Genotyping Laboratory (USDA‐ARS), following protocols described by Wang et al. (2014). Raw intensity data were processed in GenomeStudio Module Polyploid Genotyping 2.0 (Illumina Ltd.) using the custom manifest Wheat90k‐ConsAkhunovKSU‐15033654‐A. Cluster assignment and genotype calling were manually curated. Markers were filtered to exclude monomorphic sites, those with a minor allele frequency (MAF) < 0.05, and those with a call rate < 0.80. Samples with >20% missing data were removed. The final filtered dataset consisted of 30,073 SNPs. Missing genotypes were imputed, and a genomic relationship matrix was computed using the method of VanRaden (2008).

### 2.6 Genome‐wide association study

Phenotypic best linear unbiased estimates were calculated by fitting raw data to the following mixed linear model:

$$y_{ij}=\mu +g_{i}+t_{j}+\varepsilon ,$$

where *y_ij_* is the observation on the *i*th individual and *j*th replicate, *g_i_* is the fixed effect for each genotype *i*th, *t_j_* is the fixed effect of samples in each season, and *ϵ* is the residual error, assumed to be normally distributed $\varepsilon ∼N(0,\sigma^{2})$. GWAS was conducted using the BLINK model (Huang et al., 2019) implemented in GAPIT (Wang & Zhang, 2021). The analysis included the first three principal components and control AUDPC as fixed covariates to correct for population structure and disease pressure. Significance thresholds were determined using both the false discovery rate (Benjamini & Hochberg, 1995) and Bonferroni correction.

### 2.7 Gene analysis

We mapped significant SNPs to IWGSC RefSeq v2 genome (Zhu et al., 2021) using the GrainGenes genome browser (https://wheat.pw.usda.gov/GG3/) and the package GenomicRanges in R. To define candidate genes, we evaluated the genomic context of each locus: Intragenic SNPs were identified by aligning significant SNPs with gene models on the genome annotation. Additionally, to capture potential regulatory elements or nearby candidates, genes within a 20 kbp range on either side of the significant marker were delineated, and gene function was deduced based on homology to characterized genes. SNPs in linkage disequilibrium (LD) with the lead were identified using a 500 kb LD window and an *r* ^2^ ≥ 0.4. Homologs were identified using NCBI BLASTn and NCBI BLASTp (https://blast.ncbi.nlm.nih.gov/Blast.cgi) with the gene nucleotide sequence and protein sequence as the search query, respectively (*E*‐value of <10^−5^, percentage identity of ≥75%, and query cover of >50%).

## 3 RESULTS

### 3.1 STB disease phenotyping

**FIGURE 1:**
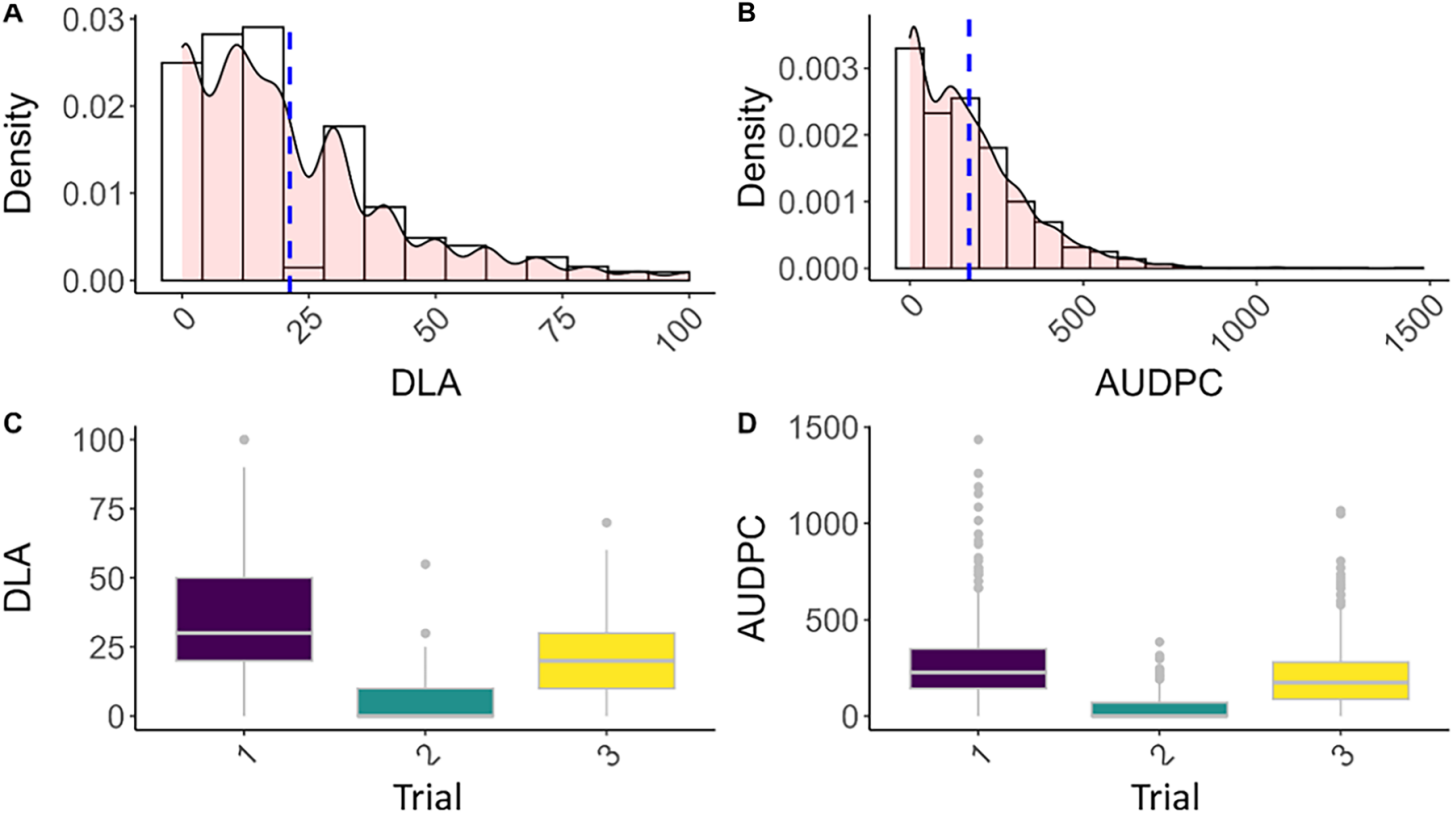
Distributions of Septoria tritici blotch severity metrics. (A) Histogram with overlaid density plot showing DLA at 28 days post‐inoculation (dpi) across all seasons. The dashed line marks the overall mean. (B) Histogram and density plot for area under the disease progress curve (AUDPC) across seasons. (C) Boxplot of the distribution of disease severity by season. (D) Boxplot of AUDPC by season.

Disease severity at 28 dpi (DLA), ranged from 0% to 100% across all seasons and varieties, with an overall mean of 21%. Trial means differed markedly: disease was moderate in trials 1 and 3 (38% and 20%) and very low in trial 2 (5%). The lower severity in trial 2 coincided with cooler temperatures at inoculation and during the growing period, consistent with reduced infection and symptom development under suboptimal conditions. AUDPC followed the same pattern, with lowest value in trial 2 (43), intermediate in trial 3 (198), and highest in trial 1 (274). Seasonal variation and the overall distribution of observations are shown in Figure 1. AUDPC and DLA were strongly correlated (Pearson's *r* = 0.86), indicating that a single late time point captured much of the variation in disease progression (Figure 1).

**FIGURE 2:**
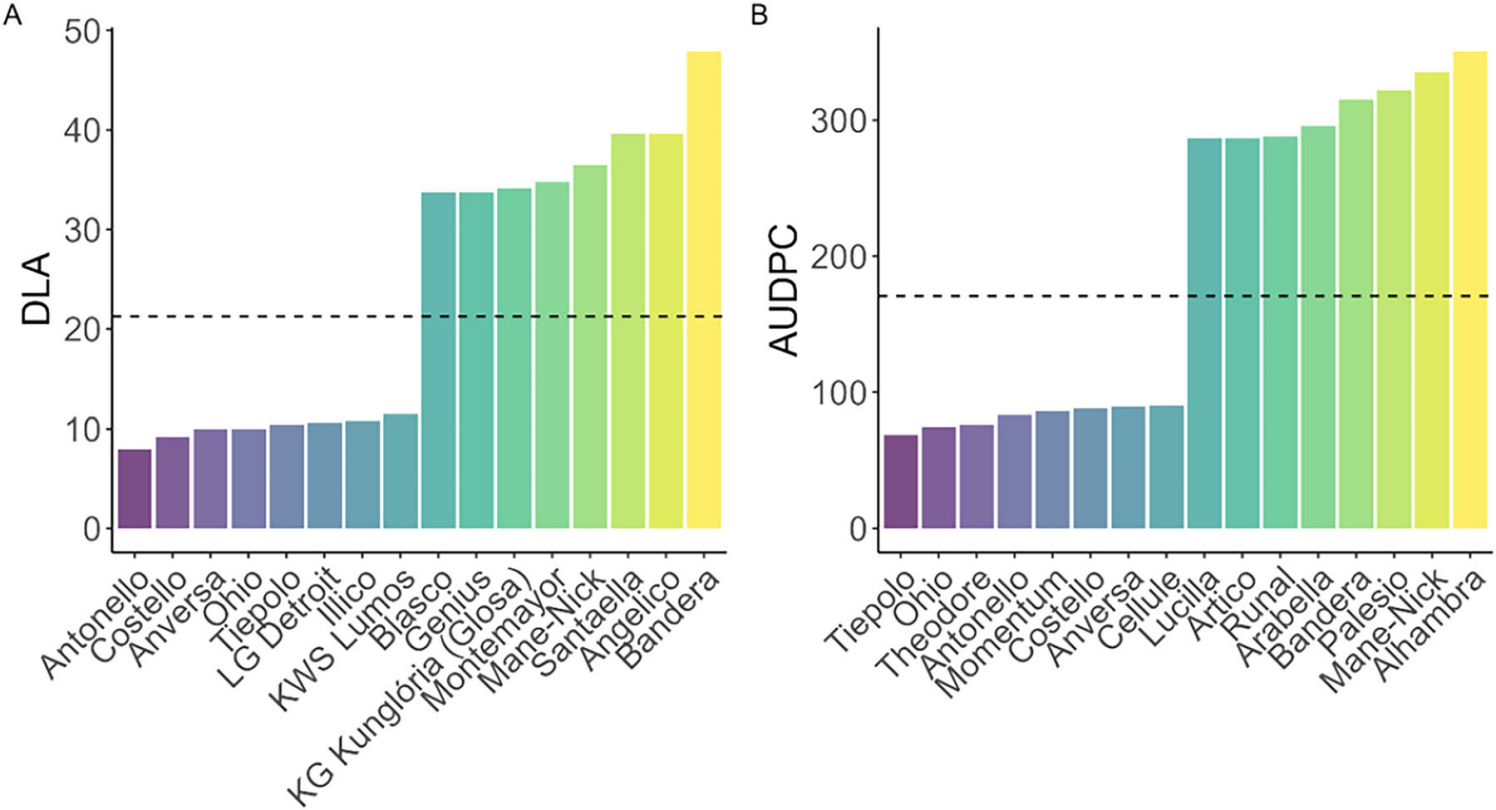
Extremes of Septoria tritici blotch (STB) response across seasons. (A) Cultivars in the lowest and highest 5% for diseased leaf area (DLA) at 28 days post‐inoculation (dpi), averaged across seasons. (B) Cultivars in the lowest and highest 5% for area under the disease progress curve (AUDPC), averaged across seasons. Bars show cultivar means; the dashed line marks the overall mean across all cultivars and seasons.

Among the cultivars, Bandera showed the highest mean DLA (48%) and one of the highest AUDPC values (315), whereas ‘Antonello’ had the lowest DLA (8%) and one of the lowest AUDPC values (83); the overall extremes for AUDPC were Alhambra (351) and Tiepolo (69) (Figure 2).

**FIGURE 3:**
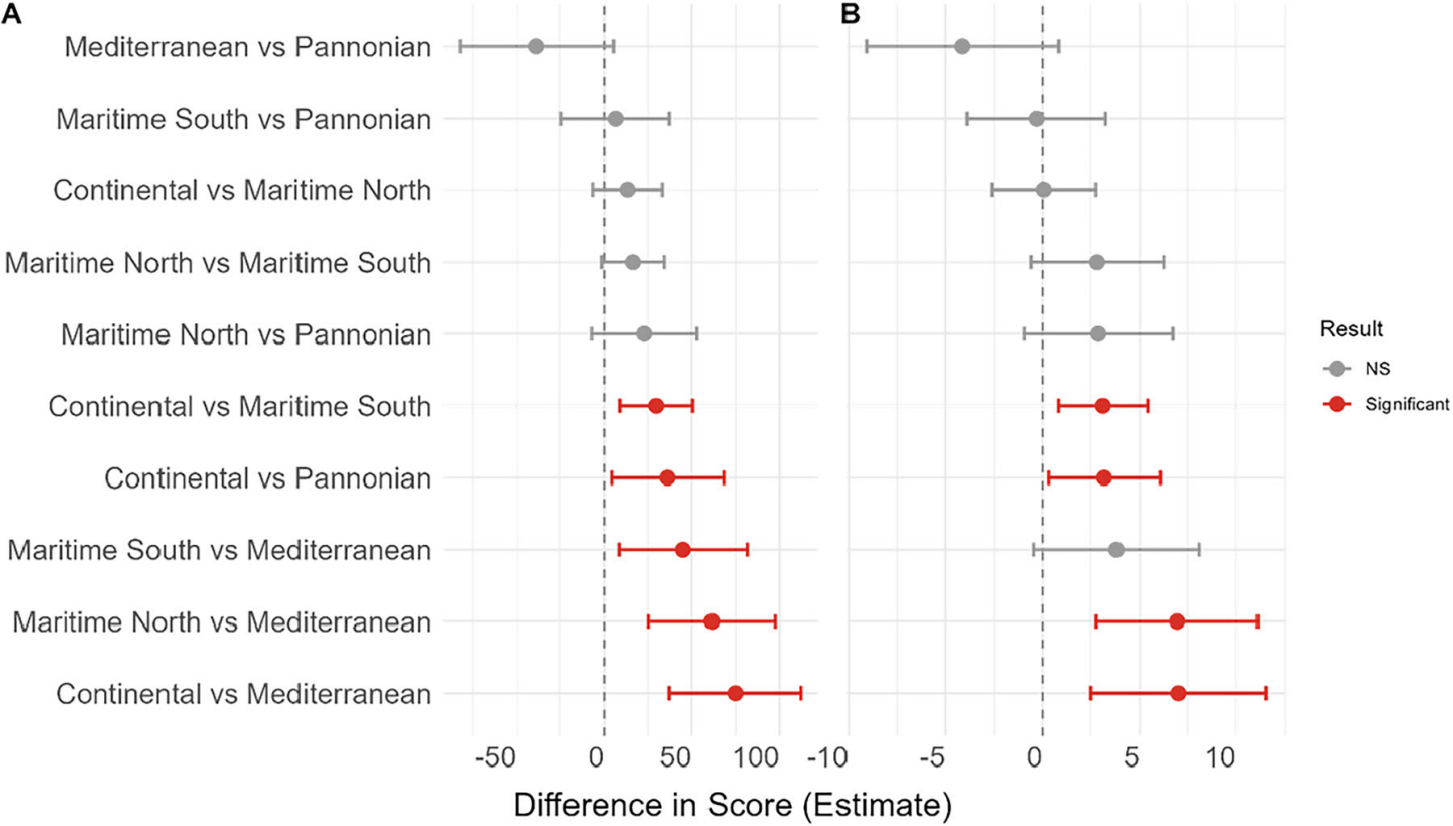
Pairwise comparisons of disease severity between agro‐climatic zones (AGZs). Plots display the mean difference in (A) area under the disease progress curve (AUDPC) and (B) diseased leaf area (DLA) for each AGZ pair. Dots represent the estimated difference in means, and horizontal bars indicate the 95% confidence intervals (CIs). Comparisons are considered statistically significant (*p* < 0.05) if the 95% CI does not cross the zero line (dashed vertical line) and are highlighted in red. Analysis performed using Welch's analysis of variance (ANOVA) with Games–Howell post hoc tests to account for unequal variances.

By AGZ, Mediterranean varieties were most affected (mean DLA = 26%; AUDPC = 221), while Continental and Maritime North showed the lowest mean DLA (both = 19%); Continental also had the lowest mean AUDPC (146) (Figure 3).

### 3.2 GWAS analysis

**FIGURE 4:**
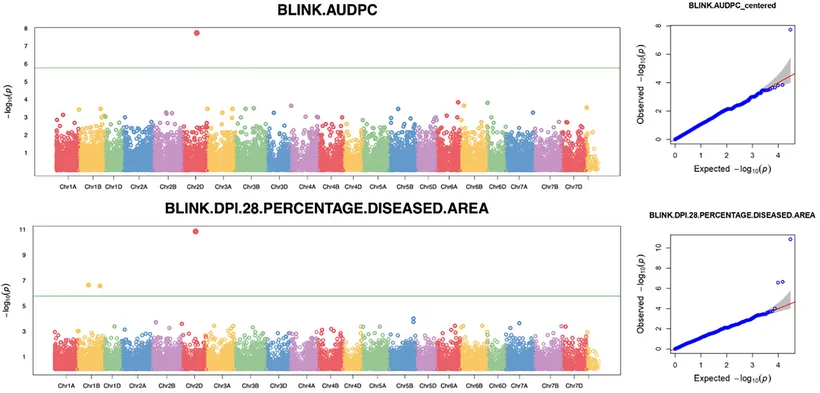
Genome‐wide association study (GWAS) results for Septoria tritici blotch (STB) resistance. Manhattan plots (left column) and quantile–quantile (Q–Q) plots (right column) generated using the BLINK model. Top panels: Area under the disease progress curve (AUDPC). Bottom panels: Percentage diseased leaf area at 28 days post‐inoculation (dpi). In Manhattan plots, single‐nucleotide polymorphisms (SNPs) are ordered physically across wheat chromosomes; the horizontal green line marks the Bonferroni‐corrected significance threshold (*α* = 0.05). In Q–Q plots, observed versus expected −log10*p*values are shown, with the red diagonal line representing the null hypothesis and gray shading indicating the 95% confidence interval. Points above the threshold correspond to the peak SNPs on 2D (IWB5774) and 1B (IWB72742 and IWB11406).

**FIGURE 5:**
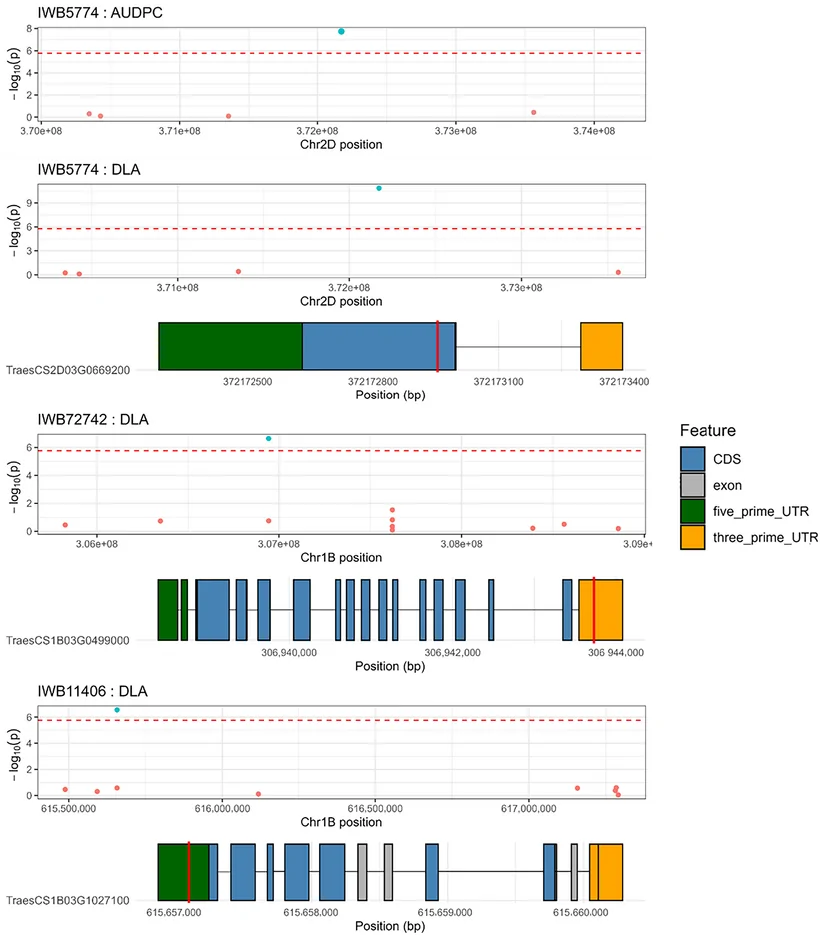
Manhattan plots showing single‐nucleotide polymorphisms (SNPs) ± 2 Mb of genome‐wide association study (GWAS)‐derived significant SNPs for both area under the disease progress curve (AUDPC) and diseased leaf area (DLA). Red dashes indicate Bonferroni‐corrected −log10(*p*) significance threshold. Also included are gene tracks for each gene associated with significant SNPs. Significant SNP position within the gene is indicated by red line. Features are color‐coded, with black lines showing introns. Position on the chromosome is shown on the *x*‐axis.

**FIGURE 6:**
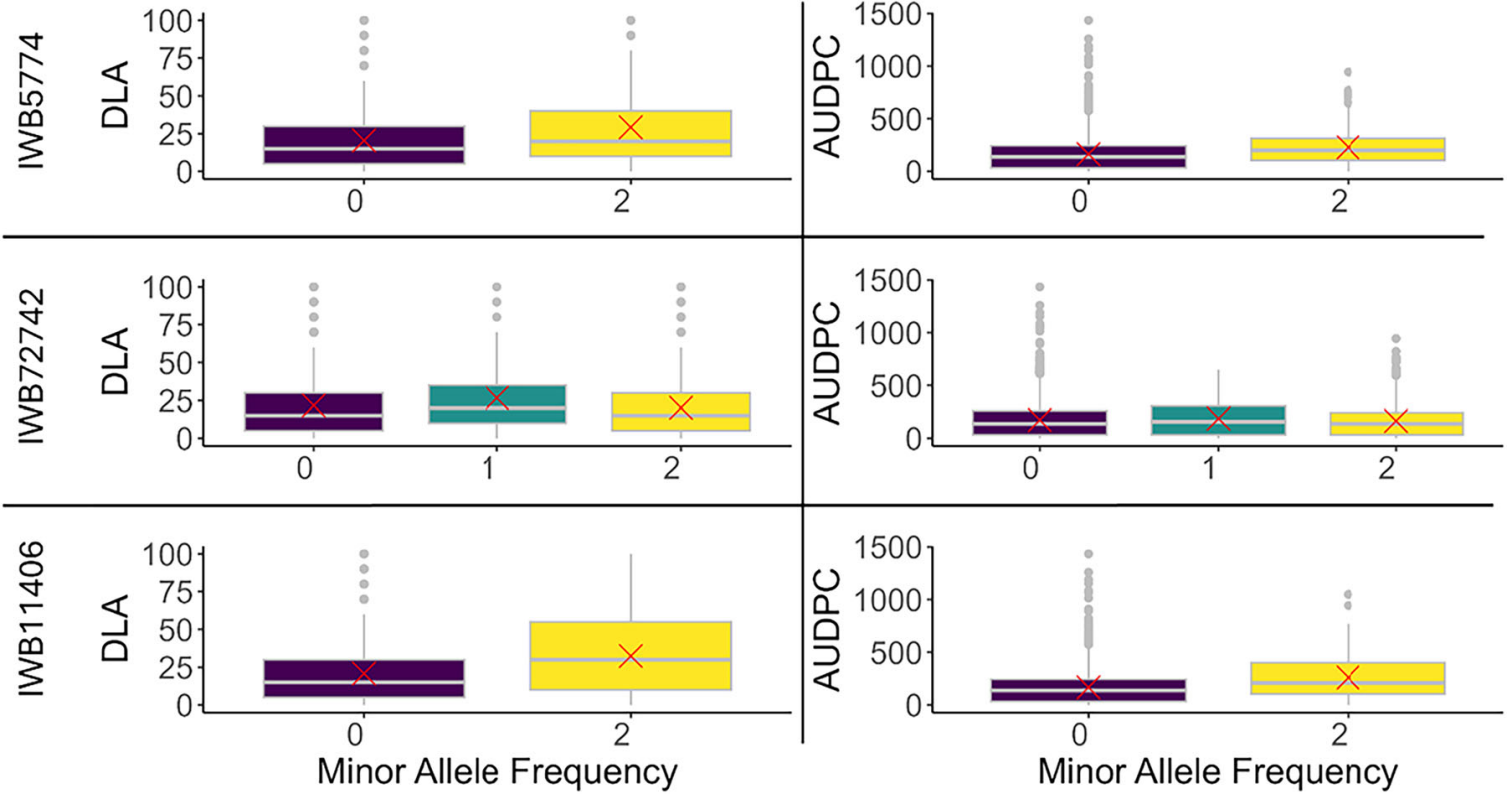
Boxplot showing spread of phenotypic trait values versus allele frequency per cultivar for each genome‐wide association study (GWAS)‐derived significant marker. Red crosses indicate mean values for that combination. AUDPC, area under the disease progress curve; DLA, diseased leaf area.

**FIGURE 7:**
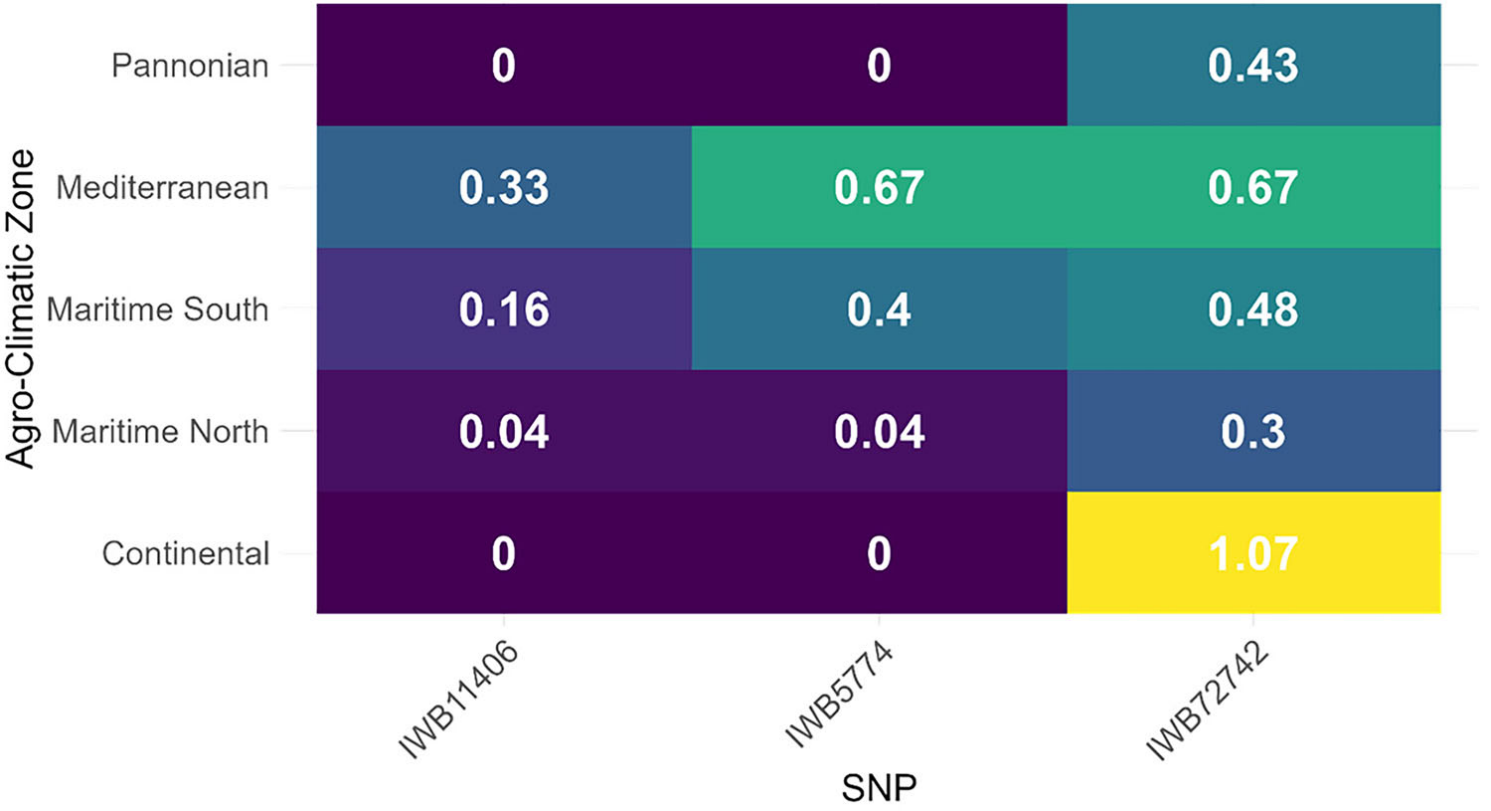
Allele frequency by agro‐climatic zone (AGZ) at significant genome‐wide association study (GWAS) single‐nucleotide polymorphisms (SNPs). Heat map shows the mean frequency of the alternate allele for each SNP (columns: IWB11406, IWB5774, and IWB72742) within each AGZ (rows). Frequency was coded per cultivar as 0 (absent), 1 (heterozygous), or 2 (homozygous) and averaged across cultivars in the zone (*n* = 21, 54, 52, 12, and 12 for Continental, Maritime North, Maritime South, Mediterranean, and Pannonian, respectively). Larger values indicate greater prevalence of the alternate allele in that zone.

Genome‐wide association scans identified three quantitative trait nucleotides significantly associated with STB resistance across models and environments (Table 1; Figures 4 and 5). (Table 1; Figures 4 and 5). LD analysis detected no additional SNPs in strong LD with these lead markers, indicating highly localized signals (Figures 4 and 5). On chromosome 2D, marker *IWB5774* significantly associated with both traits, increasing susceptibility (DLA: *β* = +15.13, *p* = 4.19 × 10^−^^7^; AUDPC: *β* = +2.28, *p* = 5.45 × 10^−^^4^). This marker maps within the coding sequence of *TraesCS2D03G0669200* (proximal to *TraesCS2D03G0669100LC*). Two distinct signals were identified on chromosome 1B. IWB72742 was negatively associated with DLA (*β* = −1.09, *p* = 2.71 × 10^−^^3^), indicating a resistance‐enhancing effect. This SNP is located in the 3′ untranslated region (UTR) of *TraesCS1B03G0499000*, which encodes a WDR91‐like WD‐repeat protein implicated in endomembrane trafficking. Conversely, *IWB11406* was positively associated with DLA and is located in the 5′ UTR of *TraesCS1B03G1027100*, encoding a nucleosome assembly protein (NAP1) involved in chromatin remodeling (Table 1; Figures 5 and 6). Allele frequencies for the significant markers varied by agro‐ecological zone (Figure 7), with MAFs of 0.103 (*IWB5774*), 0.262 (*IWB72742*), and 0.048 (*IWB11406*) in the full panel. Collectively, these associations highlight candidate genes involved in specialized metabolism, vesicle trafficking, and stress‐responsive gene regulation as targets for functional validation (Figure 7).

**TABLE 1:** Genome‐wide associated single‐nucleotide polymorphisms (SNPs) for STB response across seasons. For each SNP, the table reports chromosome (Chr) and physical position (bp; IWGSC RefSeq v2), association *p* value from BLINK, minor allele frequency (MAF) in the 151‐variety panel, the additive effect (β) of the minor allele, and the associated trait (diseased leaf area [DLA] or area under the disease progress curve [AUDPC]).

SNP	Chr:Pos (RefSeq v2)^a^	Gene model	Genic context	Annotation (common name)	Putative pathway/rationale	Associated trait(s) and effect (β)^b^	MAF
IWB5774	Chr2D: 372,172,954	TraesCS2D03G0669200^d^	Coding (synonymous A > G)	Uncharacterized; high similarity to TaBx3D	Benzoxazinoid (DIBOA) biosynthesis; defense‐related	DLA +15.13; AUDPC +2.281^c^	0.103
IWB72742	Chr1B: 306,943,730	TraesCS1B03G0499000 (WDR91)	3′ UTR	WD40 repeat protein (Rab7‐associated)	Endomembrane trafficking/stress signaling	DLA −1.09	0.262
IWB11406	Chr1B: 615,657,092	TraesCS1B03G1027100 (NAP1)	5′ UTR	Nucleosome assembly protein 1	Chromatin remodeling/defense response	DLA +2.216	0.048

*Note*: Positive β indicates increased disease (higher DLA or AUDPC); negative β indicates decreased disease. Significance thresholds and covariates are detailed in Methods.

Abbreviation: UTR, untranslated region.

^a^ Chromosome and positions refer to IWGSC RefSeq v2.

^b^ Effects (β) are additive minor‐allele effects from the GWAS model.

^c^ Units are percentage points for DLA and AUDPC units for AUDPC.

^d^ IWB5774 lies ∼2.5 kb downstream of TraesCS2D03G0669100LC.

## 4 DISCUSSION

We profiled seedling‐stage response to STB in 151 European winter wheat varieties across three polytunnel trials and found wide phenotypic dispersion for both late severity (DLA at 28 dpi) and disease progress (AUDPC), as expected for a genetically and geographically diverse panel grown under variable environments. There was high variation across the three trials, as is often typical for STB under such non‐controlled polytunnel conditions, with trial 2 having particularly low disease levels in a season of unusually low temperature and trial 1 having the highest disease pressure in a more STB‐favorable climatic season. STB infection is most severe under high humidity and temperatures of 18°C–22°C (Chungu et al., 2001; Holmes & Colhoun, 1974).

From joint ranking of DLA, AUDPC, and a composite resistance index, five cultivars (Antonello, Anversa, Costello, Ohio, and Tiepolo) consistently exhibited robust resistance, while cvs. Bandera and Mane‐Nick were most susceptible. Excluding the low‐disease season preserved these rankings and added Genius and Palesio among the most susceptible. Pycnidia coverage, assessed in trial 1 only, correlated positively with both DLA and AUDPC, in line with prior seedling studies (Ouaja et al., 2020), yet accumulating evidence suggests partly distinct genetic controls for pycnidia formation and necrosis as well as for seedling versus adult resistance (Karisto et al., 2018; Piaskowska et al., 2021; Yang et al., 2022); future field GWAS that score both traits across the canopy would therefore be informative.

Genetic background helped to explain some varietal patterns. cv. Cellule, which carries the widely deployed wall‐associated kinase Stb16q, ranked within the lowest decile for both DLA and AUDPC, whereas cv. KWS Santiago, reported to carry Stb6, showed only moderate resistance, reinforcing that durable control benefits from stacking qualitative genes with polygenic components (Brown et al., 2015; Kildea et al., 2020; Langlands‐Perry et al., 2022; Morais et al., 2019; Yang et al., 2018). Pedigree information, however, is patchy for many modern varieties; broader transparency on gene deployment would accelerate the discovery and validation of new resistance sources.

Geography also tracked susceptibility: Mediterranean accessions exhibited the highest mean severity and AUDPC, whereas Continental and Maritime North groups were least affected. Two non‐exclusive hypotheses merit testing: historical breeding programs in warmer, drier regions may have prioritized other diseases over STB, and cooler, maritime test conditions may have accentuated vulnerabilities of Mediterranean material. Reciprocal trials at higher temperatures and with local isolates would resolve environmental and isolate‐specific effects, given the well‐documented specificity of qualitative STB resistance (Brading et al., 2002; Fones & Gurr, 2015).

GWAS implicated three loci with biologically coherent candidates. IWB5774 on 2D, the strongest and most consistent signal across traits, lies in the coding region of *TraesCS2D03G0669200*, a gene with high similarity to TaBx3D, part of the benzoxazinoid (Bx) pathway that produces DIBOA‐class metabolites linked to antifungal defense in cereals (Niemeyer, 1988; Nomura et al., 2008; Sicker et al., 2000; Søltoft et al., 2008; Święcicka et al., 2020; Zhang et al., 2022). On 1B, IWB72742 maps to the 3′ UTR of *TraesCS1B03G0499000* (WDR91), a WD40 protein and Rab7‐associated effector implicated in endomembrane trafficking and stress signaling; its negative effect on DLA suggests a resistance‐enhancing allele (El‐Esawi & Alayafi, 2019; Liu et al., 2023; Ma et al., 2024; Miller et al., 2016; Tripathy et al., 2021). A second 1B association, IWB11406, sits in the 5′ UTR of *TraesCS1B03G1027100* (NAP1), a chromatin chaperone with documented roles in pathogen responses and viral challenge (Barna et al., 2018; Feng et al., 2023). The identification of TaBx3D‐like, WDR91, and NAP1 highlights the potential value of these loci for resistance breeding. Although functional validation is still required, the association of SNPs within genes linked to secondary metabolism, vesicle trafficking, and chromatin remodeling suggests that multiple, complementary biological processes may contribute to variation in STB resistance in this panel. From a breeding perspective, the intragenic markers identified here provide practical entry points for stacking distinct components of quantitative resistance. Selecting favorable alleles that enhance chemical defense (via benzoxazinoid pathways) together with improved cellular signaling or chromatin regulation (via WDR91 and NAP1) may offer a route to generating more durable, multi‐layered resistance in elite wheat cultivars.

The molecular placement of two signals in UTRs (IWB72742 and IWB11406) (Figure 5) raises testable hypotheses. Although many GWAS hits tag nearby causal variants through LD (Li et al., 2009; Kichaev & Pasaniuc, 2015), UTR variants can themselves regulate mRNA stability, localization, and translation and are known to influence disease resistance in cereals (Mayr, 2019; Xu et al., 2023; Zhang et al., 2019). Priorities include fine‐mapping and haplotype resolution around each peak, allele‐specific expression and reporter assays to test UTR effects, and targeted editing to evaluate causality. IWB5774 is a synonymous A > G substitution; despite not altering the amino acid sequence, synonymous changes can modulate mRNA structure and translation kinetics and thereby protein abundance and function (Nackley et al., 2006; Rauscher & Ignatova, 2018; Sauna & Kimchi‐Sarfaty, 2011). Together, benzoxazinoid metabolism, endomembrane trafficking, and chromatin remodeling emerge as convergent routes to STB resistance that warrant functional validation.

This study has limitations: polytunnel conditions precluded strict environmental control; seedling screens may not capture adult‐plant resistance; the mixed‐isolate inoculum improves breadth but complicates isolate‐specific inference; and one association (IWB11406) had low minor‐allele frequency, advocating cautious interpretation. Notably, we did not detect associations with previously characterized *Stb* genes. This is likely due to the high frequency of such genes (e.g., *Stb6*) in the population, which limits the genotypic and phenotypic variance required for GWAS detection (Saintenac et al., 2018). Furthermore, a mixed inoculum can mask gene‐for‐gene interactions, reducing the likelihood that such genes will emerge as significant. Finally, many documented QTLs derive from biparental crosses of exotic material and are likely rare enough that they are removed at the MAF filtering stage or lack the statistical power necessary for detection. Even so, the combination of multi‐season phenotyping and robust GWAS nominates three tractable loci that can be immediately leveraged in breeding, either as fixed diagnostic markers or within genomic selection pipelines, while mechanistic work tests causality and stage specificity. Ultimately, pyramiding major genes such as Stb16q with validated minor‐effect alleles from these pathways, and benchmarking under diverse environments and isolate panels, should improve durability and reduce reliance on fungicides (Cheval et al., 2017; Fones & Gurr, 2015; Talas et al., 2024; Torriani et al., 2015; Wieczorek et al., 2015). We quantified seedling responses to STB in 151 European winter wheats and mapped three loci associated with reduced or increased disease: IWB5774 (2D; TaBx3D‐like), IWB72742 (1B; WDR91), and IWB11406 (1B; NAP1). These candidates converge on benzoxazinoid metabolism, endomembrane trafficking, and chromatin remodeling, pointing to complementary routes for strengthening quantitative resistance. Allele distributions and cultivar rankings underscore the polygenic, environment‐sensitive nature of STB defense. The markers provide immediate tools for selection and for stacking with major genes such as *Stb16q*. Priorities now are fine‐mapping and haplotype resolution, allele‐specific expression and UTR functional assays, targeted editing to test causality, and field validation across growth stages and isolate panels to ensure durability.

## AUTHOR CONTRIBUTIONS

**Conor Copeland**: Data curation; formal analysis; investigation; visualization; writing—original draft. **Julio Isidro y Sánchez**: Data curation; formal analysis; investigation; visualization; writing—review and editing. **Humberto Fanelli**: Data curation; formal analysis; investigation; writing—review and editing. **Fiona M. Doohan**: Conceptualization; funding acquisition; project administration; supervision; writing—review and editing.

## CONFLICT OF INTEREST STATEMENT

The authors declare no conflicts of interest.

## Supporting information




**Figure S1** Map showing the locations of different agro‐climatic zones in Europe.

**Figure S2** Phlyogenetic tree showing genetic distances between included wheat varieties.

**Table S1** List of included varieties and their agroclimatic zone of origin.

**Table S2** NCBI BLASTn hits for each of the genes containing GWAS‐derived significant markers (Query cover > 70%, E‐Value, Percentage identity > 90%).

**Table S3** NCBI BLASTp hits for each of the genes containing GWAS‐derived significant markers markers (Query cover > 70%, E‐Value, Percentage identity > 90%).

## Data Availability

All data generated and analyzed in this study are available online at https://github.com/conorcopeland3/GWAS2025.
