# Supplementary material for: Phenotypic and genetic resistance to Septoria blotch disease in European wheat varieties
Source: Plant Genome. 2026 Mar 30;19(2):e70237. doi: 10.1002/tpg2.70237 (PMC13034100; doi:10.1002/tpg2.70237)
Supplement: Supplementary file 1 [file TPG2-19-e70237-s002.pdf]

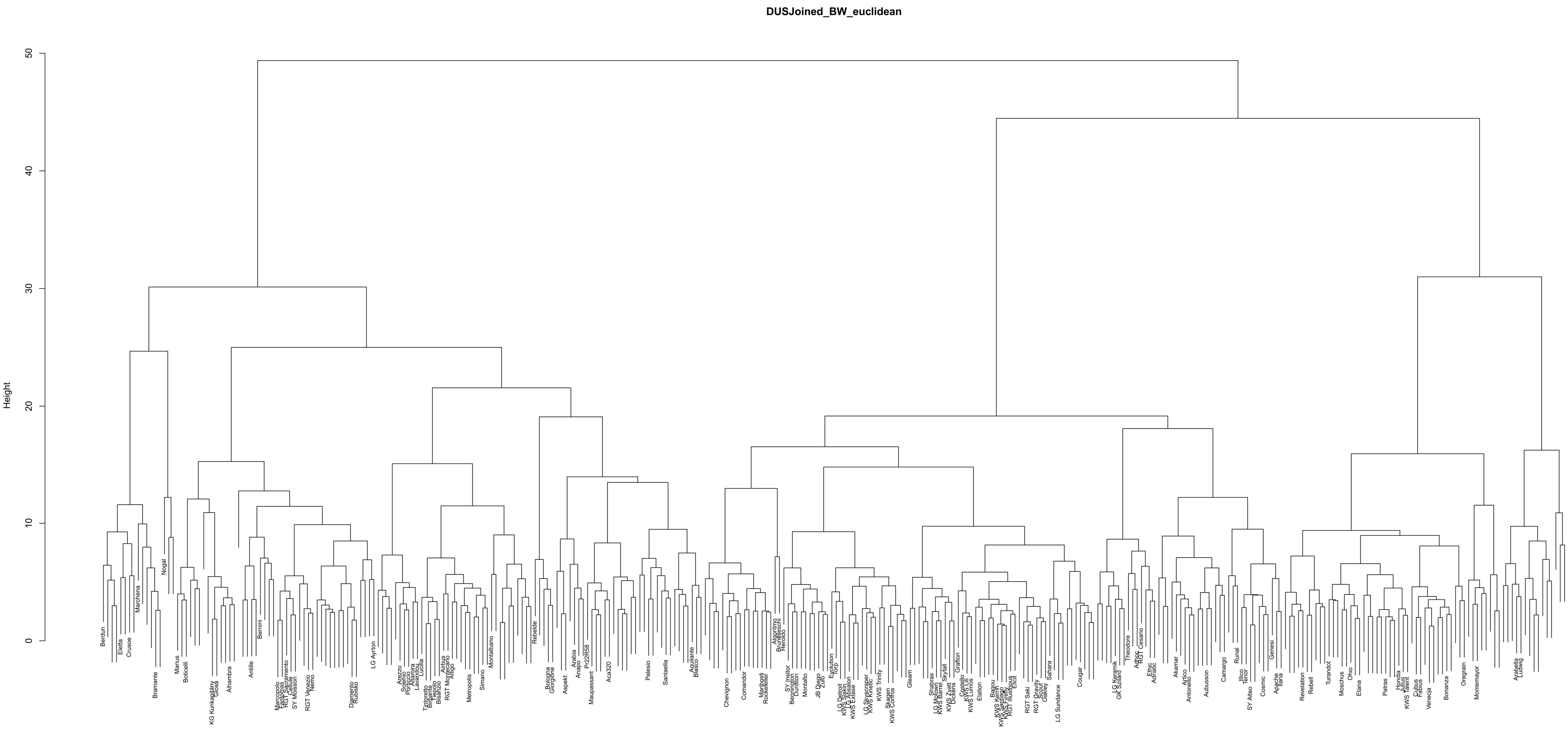
DUSJoined_BW_euclidean
Height
0
10
20
30
40
50
