# Supplementary material for: Phenotypic and genetic resistance to Septoria blotch disease in European wheat varieties
Source: Plant Genome. 2026 Mar 30;19(2):e70237. doi: 10.1002/tpg2.70237 (PMC13034100; doi:10.1002/tpg2.70237)
Supplement: Supplementary file 3 [file TPG2-19-e70237-s001.docx]

Table S3: NCBI BLASTp hits for each of the genes containing GWAS-derived significant markers markers (Query cover >70%, E-Value, Percentage identity >90%).

| **Marker** | **Ensembl Gene ID** | **NCBI Gene ID** | **NCBI Accession** | **Description** | **Query Cover** | **E value** | **Per. ident** | **NCBI Accession** |
| --- | --- | --- | --- | --- | --- | --- | --- | --- |
| IWB5774 | TraesCS2D03G0669200 | LOC123053206 | XP_044332574 | uncharacterized protein [Aegilops tauschii subsp. strangulata] | 100% | 3E-77 | 99.17 | XP_020169834.1 |
|  |  |  |  | uncharacterized protein LOC125537981 [Triticum urartu] | 100% | 3E-76 | 98.35 | XP_048557254.1 |
|  |  |  |  | hypothetical protein CFC21_017267 [Triticum aestivum] | 100% | 1E-74 | 96.69 | KAF7001645.1 |
|  |  |  |  | uncharacterized protein LOC119355043 [Triticum dicoccoides] | 100% | 2E-74 | 96.69 | XP_037477721.1 |
|  |  |  |  | unnamed protein product [Triticum turgidum subsp. durum] | 100% | 5E-74 | 95.87 | VAH32092.1 |
|  |  |  |  | uncharacterized protein LOC123426612 [Hordeum vulgare subsp. vulgare] | 100% | 2E-73 | 97.52 | XP_044966414.1 |
|  |  |  |  | uncharacterized protein [Triticum aestivum] | 100% | 3E-73 | 96.69 | XP_044324330.1 |
|  |  |  |  | uncharacterized protein LOC119364171 [Triticum dicoccoides] | 100% | 5E-73 | 96.69 | XP_037485495.1 |
|  |  |  |  | hypothetical protein ZWY2020_056829 [Hordeum vulgare] | 100% | 2E-72 | 97.52 | KAI5015439.1 |
|  |  |  |  | uncharacterized protein [Aegilops tauschii subsp. strangulata] | 100% | 2E-71 | 93.5 | XP_020182493.1 |
|  |  |  |  | hypothetical protein VPH35_076395 [Triticum aestivum] | 74% | 6E-39 | 95.51 | XBH54016.1 |
|  |  |  |  | hypothetical protein VPH35_037533 [Triticum aestivum] | 74% | 3E-29 | 94.38 | XBI55804.1 |
|  |  |  |  | hypothetical protein VPH35_053145 [Triticum aestivum] | 72% | 2E-28 | 90.8 | XBI29061.1 |
|  |  |  |  | unnamed protein product [Triticum turgidum subsp. durum] | 70% | 2E-27 | 90.59 | VAI92392.1 |
|  |  |  |  | unnamed protein product [Triticum turgidum subsp. durum] | 70% | 3E-26 | 94.12 | VAI01112.1 |
| IWB72742 | TraesCS1B03G0499000 | LOC123121052 | XP_044396927 | WD repeat-containing protein 91 homolog [Triticum dicoccoides] | 100% | 0 | 99.52 | XP_037450849.1 |
|  |  |  |  | unnamed protein product [Triticum turgidum subsp. durum] | 100% | 0 | 99.36 | VAH16247.1 |
|  |  |  |  | WD repeat-containing protein 91 homolog [Triticum aestivum] | 100% | 0 | 98.56 | XP_044449158.1 |
|  |  |  |  | uncharacterized protein [Aegilops tauschii subsp. strangulata] | 100% | 0 | 98.39 | XP_020173178.1 |
|  |  |  |  | WD repeat-containing protein 91 homolog [Triticum aestivum] | 100% | 0 | 97.28 | XP_044324141.1 |
|  |  |  |  | WD repeat-containing protein 91 homolog [Triticum urartu] | 100% | 0 | 96.8 | XP_048527112.1 |
|  |  |  |  | hypothetical protein CFC21_001596 [Triticum aestivum] | 100% | 0 | 96.64 | KAF6983404.1 |
|  |  |  |  | WD repeat-containing protein 91 homolog [Triticum dicoccoides] | 100% | 0 | 96.64 | XP_037406302.1 |
|  |  |  |  | WD repeat-containing protein 91 homolog [Hordeum vulgare subsp. vulgare] | 100% | 0 | 96.16 | XP_044970571.1 |
|  |  |  |  | unnamed protein product [Triticum turgidum subsp. durum] | 100% | 0 | 96 | VAH04204.1 |
|  |  |  |  | hypothetical protein CFC21_006395 [Triticum aestivum] | 100% | 0 | 95.18 | KAF6988992.1 |
|  |  |  |  | hypothetical protein VPH35_008073 [Triticum aestivum] | 95% | 0 | 98.65 | XBJ16439.1 |
|  |  |  |  | hypothetical protein CFC21_011224 [Triticum aestivum] | 100% | 0 | 93.74 | KAF6994553.1 |
|  |  |  |  | hypothetical protein VPH35_008073 [Triticum aestivum] | 95% | 0 | 98.14 | XBJ16438.1 |
|  |  |  |  | hypothetical protein CFC21_001596 [Triticum aestivum] | 100% | 0 | 91.84 | KAF6983403.1 |
|  |  |  |  | hypothetical protein VPH35_001954 [Triticum aestivum] | 95% | 0 | 95.29 | XBJ23967.1 |
|  |  |  |  | hypothetical protein CFC21_001596 [Triticum aestivum] | 93% | 0 | 96.56 | KAF6983405.1 |
|  |  |  |  | hypothetical protein CFC21_006395 [Triticum aestivum] | 73% | 0 | 100 | KAF6988996.1 |
|  |  |  |  | hypothetical protein VPH35_014090 [Triticum aestivum] | 70% | 0 | 97.95 | XBJ08904.1 |
| IWB11406 | TraesCS1B03G1027100 | LOC123142563 | XP_044417363 | nucleosome assembly protein 1;2-like [Triticum dicoccoides] | 100% | 0 | 98.09 | [XP_037473154.1](https://www.ncbi.nlm.nih.gov/protein/XP_037473154.1?report=genbank&log$=prottop&blast_rank=2&RID=843GR51R015) |
|  |  |  |  | hypothetical protein CFC21_008498 [Triticum aestivum] | 100% | 0 | 98.89 | [KAF6991412.1](https://www.ncbi.nlm.nih.gov/protein/KAF6991412.1?report=genbank&log$=prottop&blast_rank=3&RID=843GR51R015) |
|  |  |  |  | nucleosome assembly protein 1;2 isoform X2 [Triticum dicoccoides] | 100% | 0 | 96.17 | [XP_037424287.1](https://www.ncbi.nlm.nih.gov/protein/XP_037424287.1?report=genbank&log$=prottop&blast_rank=4&RID=843GR51R015) |
|  |  |  |  | nucleosome assembly protein 1;2 [Triticum urartu] | 100% | 0 | 95.9 | [XP_048547339.1](https://www.ncbi.nlm.nih.gov/protein/XP_048547339.1?report=genbank&log$=prottop&blast_rank=5&RID=843GR51R015) |
|  |  |  |  | nucleosome assembly protein 1;2 [Hordeum vulgare subsp. vulgare] | 100% | 0 | 95.11 | [XP_044981036.1](https://www.ncbi.nlm.nih.gov/protein/XP_044981036.1?report=genbank&log$=prottop&blast_rank=6&RID=843GR51R015) |
|  |  |  |  | hypothetical protein ZWY2020_044661 [Hordeum vulgare] | 100% | 0 | 94.84 | [KAI5019773.1](https://www.ncbi.nlm.nih.gov/protein/KAI5019773.1?report=genbank&log$=prottop&blast_rank=7&RID=843GR51R015) |
|  |  |  |  | unnamed protein product [Triticum turgidum subsp. durum] | 100% | 0 | 95.63 | [VAH09921.1](https://www.ncbi.nlm.nih.gov/protein/VAH09921.1?report=genbank&log$=prottop&blast_rank=8&RID=843GR51R015) |
|  |  |  |  | nucleosome assembly protein 1;2 [Aegilops tauschii subsp. strangulata] | 100% | 0 | 95.08 | [XP_020160372.1](https://www.ncbi.nlm.nih.gov/protein/XP_020160372.1?report=genbank&log$=prottop&blast_rank=9&RID=843GR51R015) |
|  |  |  |  | hypothetical protein VPH35_016616 [Triticum aestivum] | 97% | 0 | 94.63 | [XBJ12013.1](https://www.ncbi.nlm.nih.gov/protein/XBJ12013.1?report=genbank&log$=prottop&blast_rank=10&RID=843GR51R015) |
|  |  |  |  | hypothetical protein VPH35_016616 [Triticum aestivum] | 94% | 0 | 96.22 | [XBJ12014.1](https://www.ncbi.nlm.nih.gov/protein/XBJ12014.1?report=genbank&log$=prottop&blast_rank=11&RID=843GR51R015) |
|  |  |  |  | unnamed protein product [Alopecurus aequalis] | 100% | 0 | 90.22 | [CAM0954252.1](https://www.ncbi.nlm.nih.gov/protein/CAM0954252.1?report=genbank&log$=prottop&blast_rank=12&RID=843GR51R015) |
