# Supplementary material for: Phenotypic and genetic resistance to Septoria blotch disease in European wheat varieties
Source: Plant Genome. 2026 Mar 30;19(2):e70237. doi: 10.1002/tpg2.70237 (PMC13034100; doi:10.1002/tpg2.70237)
Supplement: Supplementary file 4 [file TPG2-19-e70237-s003.docx]

Table S1: List of included varieties and their agroclimatic zone of origin.

| **Variety Name** | **Agro-Climatic Zone** |
| --- | --- |
| Aca320 | Maritime South |
| Adhoc | Maritime South |
| Adriatic | Mediterranean |
| Airbus | Pannonian |
| Akamar | Maritime South |
| Alhambra | Pannonian |
| Altamira | Maritime South |
| Altigo | Maritime North |
| Anapo | Maritime South |
| Antille | Maritime South |
| Apache | Pannonian |
| Aquilante | Maritime South |
| Arabella | Mediterranean |
| Arabia | Maritime South |
| Arezzo | Maritime South |
| Artico | Maritime South |
| Aspekt | Continental |
| Aubusson | Maritime South |
| Avatar | Maritime North |
| Bagou | Maritime North |
| Basmati | Maritime South |
| Bennington | Maritime North |
| Berdun | Maritime South |
| Bernini | Maritime South |
| Birbante | Maritime South |
| Bisanzio | Maritime South |
| Blasco | Maritime South |
| Bologna | Maritime South |
| Bonanza | Continental |
| Botticelli | Maritime South |
| Bramante | Maritime South |
| Camargo | Maritime South |
| Cellule | Pannonian |
| Chevignon | Maritime South |
| Comandor | Continental |
| Cosmic | Maritime South |
| Costello | Maritime North |
| Cougar | Maritime North |
| Crusoe | Maritime North |
| Cubus | Continental |
| Detroit | Maritime North |
| Diamento | Maritime South |
| Dickens | Maritime North |
| Donator | Pannonian |
| Dunston | Maritime North |
| Elation | Maritime North |
| Eletta | Maritime South |
| Elicit | Maritime North |
| Epson | Maritime North |
| Etana | Continental |
| Ethic | Maritime South |
| Fantomas | Mediterranean |
| Garrus | Maritime North |
| Genesi | Maritime South |
| Giorgione | Maritime South |
| Gleam | Maritime North |
| Grafton | Maritime North |
| Gravity | Maritime North |
| Hondia | Continental |
| Ilaria | Maritime South |
| Illico | Maritime South |
| Jb Diego | Maritime North |
| Julius | Continental |
| Kg Kungloria (Glosa) | Maritime South |
| Kws Barrel | Maritime North |
| Kws Conros | Maritime North |
| Kws Extase | Maritime North |
| Kws Firefly | Maritime North |
| Kws Illustrious | Maritime North |
| Kws Kerrin | Maritime North |
| Kws Kinetic | Maritime North |
| Kws Lili | Maritime North |
| Kws Lumos Rl17 | Maritime North |
| Kws Santiago | Maritime North |
| Kws Siskin | Maritime North |
| Kws Trinity | Maritime North |
| Kws Zyatt | Maritime North |
| Lavandou | Maritime South |
| Lg Absalon | Maritime South |
| Lg Keramik | Continental |
| Lg Skyscraper | Maritime North |
| Lg Spotlight | Maritime North |
| Lucilla | Maritime South |
| Ludwig | Maritime South |
| Lukullus | Pannonian |
| Marchena | Mediterranean |
| Marcopolo | Maritime South |
| Mariboss | Continental |
| Marius | Mediterranean |
| Maupasant | Mediterranean |
| Metropolis | Maritime South |
| Montalbano | Maritime North |
| Montalto | Maritime North |
| Montecarlo | Pannonian |
| Montemayor | Mediterranean |
| Moschus | Continental |
| Motown | Maritime North |
| Mv Kolo | Pannonian |
| Nemo | Maritime South |
| Nogal | Maritime South |
| Oakley | Continental |
| Ohio | Continental |
| Oregrain | Maritime South |
| Paledor | Mediterranean |
| Palesio | Maritime South |
| Patras | Continental |
| Pibrac | Maritime South |
| Porticcio | Mediterranean |
| Pr22R58 | Maritime South |
| Rebelde | Maritime South |
| Rebell | Continental |
| Reflection | Maritime North |
| Revelation | Maritime North |
| Rgt Cesario | Maritime South |
| Rgt Saki | Maritime North |
| Rgt Venezio | Pannonian |
| Rockefeller Rl17 | Maritime North |
| Rubisko | Pannonian |
| Runal | Maritime North |
| Sahara | Maritime North |
| Santaella | Mediterranean |
| Santorin | Pannonian |
| Shabras | Maritime North |
| Simano | Maritime North |
| Skagen | Continental |
| Sobred | Maritime South |
| Soledad | Maritime South |
| Solehio | Maritime South |
| Sundance | Maritime North |
| Sy Alteo | Mediterranean |
| Sy Insitor | Maritime North |
| Sy Moisson | Maritime South |
| Szilard | Pannonian |
| Tabasco | Continental |
| Taylor | Maritime South |
| Tenor | Mediterranean |
| Theodore | Maritime North |
| Tiepolo | Maritime South |
| Tintoretto | Maritime South |
| Torp | Maritime North |
| Turandot | Continental |
| Vanessa | Continental |
| Venecja | Continental |
| Viscount | Maritime North |
| W100003 Cordiale | Maritime North |
| W10043 Scout | Maritime North |
| W10093 Evolution | Maritime North |
| W10198 Skyfall | Maritime North |
| W10309 Rgt Sacramento | Continental |
| W2087 Talent | Continental |
| Zulu | Maritime North |
