# Supplementary figures and images for: Phenotypic and genetic resistance to Septoria blotch disease in European wheat varieties
Source: Plant Genome. 2026 Mar 30;19(2):e70237. doi: 10.1002/tpg2.70237 (PMC13034100; doi:10.1002/tpg2.70237)

- Maritime North
- Mediterranean
- Maritime South
- Continental
- Pannonian

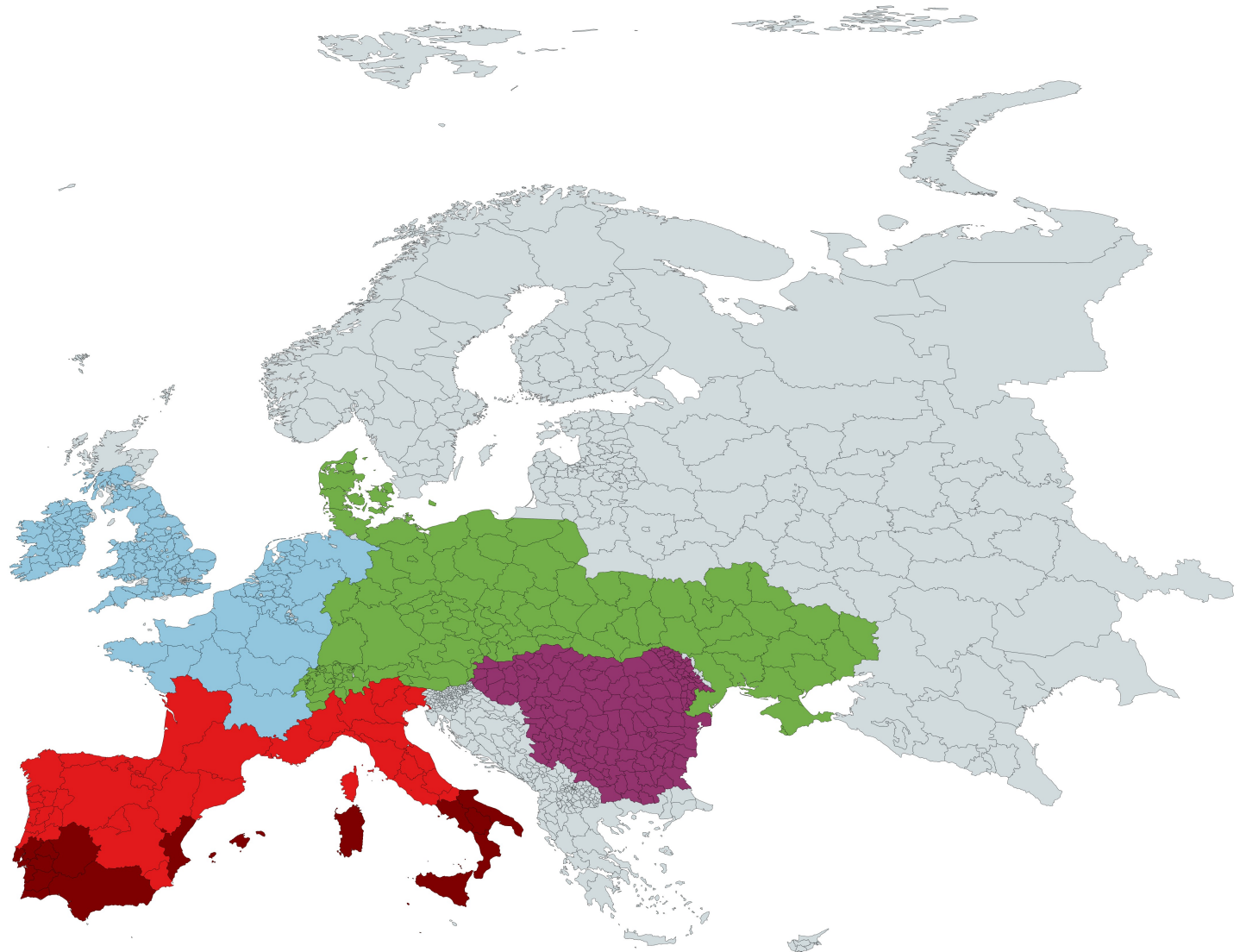

Supplement: Supplementary file 1 — Figure S1 Map showing the locations of different agro‐climatic zones in Europe. [file TPG2-19-e70237-s004.pdf]

DUSJoined\_BW\_euclidean

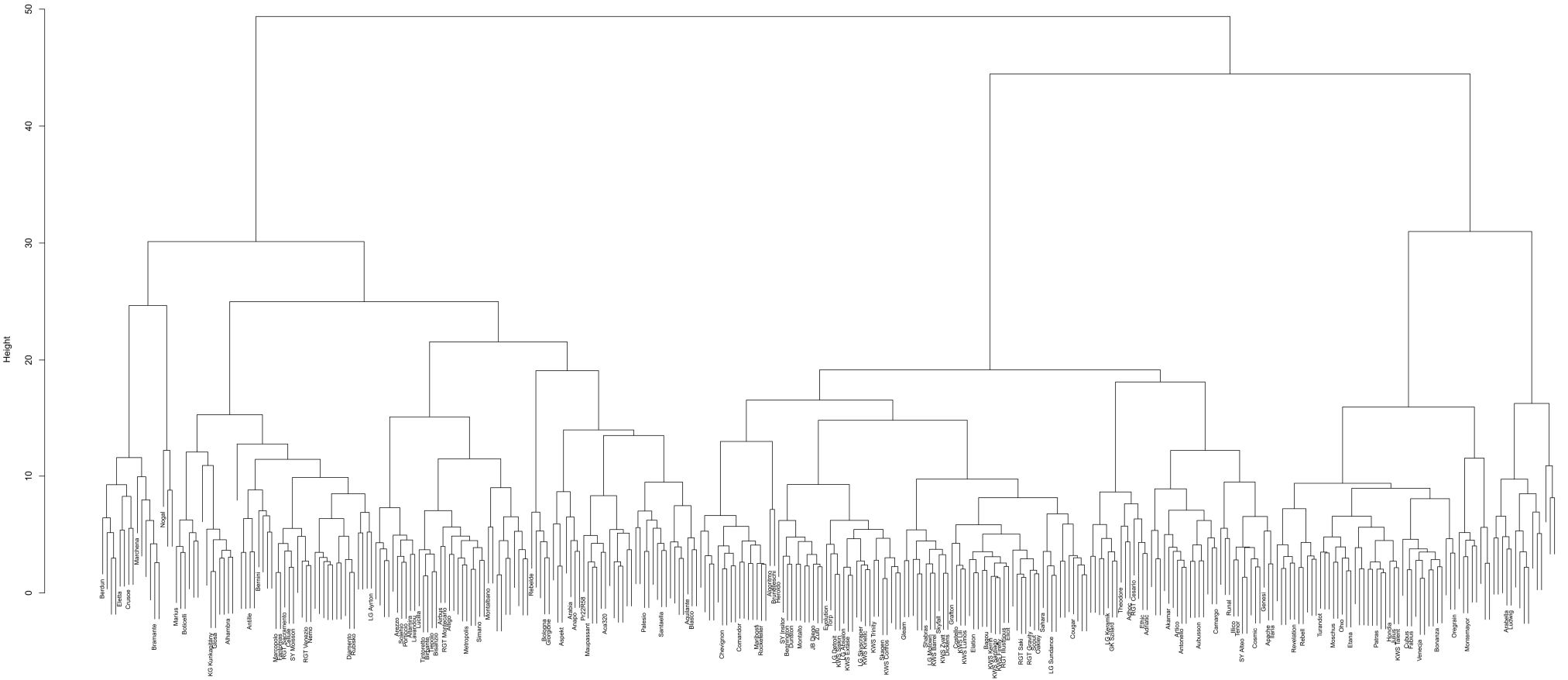

Supplement: Supplementary file 2 — Figure S2 Phlyogenetic tree showing genetic distances between included wheat varieties. [file TPG2-19-e70237-s002.pdf]
