# Supplementary material for: Phenotypic and genetic resistance to Septoria blotch disease in European wheat varieties
Source: Plant Genome. 2026 Mar 30;19(2):e70237. doi: 10.1002/tpg2.70237 (PMC13034100; doi:10.1002/tpg2.70237)
Supplement: Supplementary file 4 — Table S2 NCBI BLASTn hits for each of the genes containing GWAS‐derived significant markers (Query cover > 70%, E‐Value, Percentage identity > 90%). [file TPG2-19-e70237-s005.docx]

Table S2: NCBI BLASTn hits for each of the genes containing GWAS-derived significant markers (Query cover >70%, E-Value, Percentage identity >90%).

| **Marker** | **Ensembl Gene ID** | **NCBI Gene ID** | **NCBI Accession** | **Description** | **Query Cover** | **E value** | **Per. ident** | **NCBI Accession** |
| --- | --- | --- | --- | --- | --- | --- | --- | --- |
| IWB5774 | TraesCS2D03G0669200 | LOC123053206 | XM_044476639.1 | PREDICTED: Aegilops tauschii subsp. strangulata uncharacterized protein (LOC109755330), mRNA | 93% | 0 | 99.79 | [XM_020314245.4](https://www.ncbi.nlm.nih.gov/nucleotide/XM_020314245.4?report=genbank&log$=nucltop&blast_rank=2&RID=842YVRW7014) |
|  |  |  |  | PREDICTED: Triticum aestivum uncharacterized protein (LOC123045366), mRNA | 92% | 0 | 91.63 | [XM_044468395.1](https://www.ncbi.nlm.nih.gov/nucleotide/XM_044468395.1?report=genbank&log$=nucltop&blast_rank=3&RID=842YVRW7014) |
|  |  |  |  | PREDICTED: Triticum urartu uncharacterized LOC125537981 (LOC125537981), transcript variant X1, mRNA | 88% | 0 | 90.62 | [XM_048701297.1](https://www.ncbi.nlm.nih.gov/nucleotide/XM_048701297.1?report=genbank&log$=nucltop&blast_rank=4&RID=842YVRW7014) |
|  |  |  |  | PREDICTED: Triticum urartu uncharacterized LOC125537985 (LOC125537985), transcript variant X1, mRNA | 88% | 0 | 90.62 | [XM_048701299.1](https://www.ncbi.nlm.nih.gov/nucleotide/XM_048701299.1?report=genbank&log$=nucltop&blast_rank=5&RID=842YVRW7014) |
|  |  |  |  | Triticum aestivum cDNA, clone: SET1_L13, cultivar: Chinese Spring | 70% | 0 | 92.5 | [AK332036.1](https://www.ncbi.nlm.nih.gov/nucleotide/AK332036.1?report=genbank&log$=nucltop&blast_rank=6&RID=842YVRW7014) |
| IWB72742 | TraesCS1B03G0499000 | LOC123121052 | XM_044540992.1 | PREDICTED: Triticum dicoccoides WD repeat-containing protein 91 homolog (LOC119321125), mRNA | 98% | 0 | 99.15 | [XM_037594952.1](https://www.ncbi.nlm.nih.gov/nucleotide/XM_037594952.1?report=genbank&log$=nucltop&blast_rank=2&RID=842NJS9E015) |
|  |  |  |  | PREDICTED: Aegilops tauschii subsp. strangulata uncharacterized protein (LOC109758726), transcript variant X1, mRNA | 98% | 0 | 97.28 | [XM_020317589.4](https://www.ncbi.nlm.nih.gov/nucleotide/XM_020317589.4?report=genbank&log$=nucltop&blast_rank=3&RID=842NJS9E015) |
|  |  |  |  | PREDICTED: Triticum aestivum WD repeat-containing protein 91 homolog (LOC123181015), transcript variant X1, mRNA | 98% | 0 | 97 | [XM_044593223.1](https://www.ncbi.nlm.nih.gov/nucleotide/XM_044593223.1?report=genbank&log$=nucltop&blast_rank=4&RID=842NJS9E015) |
|  |  |  |  | PREDICTED: Triticum urartu WD repeat-containing protein 91 homolog (LOC125506304), mRNA | 97% | 0 | 95.66 | [XM_048671155.1](https://www.ncbi.nlm.nih.gov/nucleotide/XM_048671155.1?report=genbank&log$=nucltop&blast_rank=5&RID=842NJS9E015) |
|  |  |  |  | PREDICTED: Aegilops tauschii subsp. strangulata uncharacterized protein (LOC109758726), transcript variant X2, mRNA | 98% | 0 | 98.15 | [XM_073510952.1](https://www.ncbi.nlm.nih.gov/nucleotide/XM_073510952.1?report=genbank&log$=nucltop&blast_rank=6&RID=842NJS9E015) |
|  |  |  |  | PREDICTED: Triticum aestivum WD repeat-containing protein 91 homolog (LOC123045234), mRNA | 95% | 0 | 95.84 | [XM_044468206.1](https://www.ncbi.nlm.nih.gov/nucleotide/XM_044468206.1?report=genbank&log$=nucltop&blast_rank=7&RID=842NJS9E015) |
|  |  |  |  | PREDICTED: Triticum dicoccoides WD repeat-containing protein 91 homolog (LOC119268705), transcript variant X1, mRNA | 94% | 0 | 95.67 | [XM_037550405.1](https://www.ncbi.nlm.nih.gov/nucleotide/XM_037550405.1?report=genbank&log$=nucltop&blast_rank=8&RID=842NJS9E015) |
|  |  |  |  | PREDICTED: Hordeum vulgare subsp. vulgare WD repeat-containing protein 91 homolog (LOC123430797), transcript variant X1, mRNA | 94% | 0 | 94.37 | [XM_045114636.1](https://www.ncbi.nlm.nih.gov/nucleotide/XM_045114636.1?report=genbank&log$=nucltop&blast_rank=9&RID=842NJS9E015) |
|  |  |  |  | PREDICTED: Triticum dicoccoides WD repeat-containing protein 91 homolog (LOC119268705), transcript variant X2, mRNA | 87% | 0 | 95.67 | [XM_037550413.1](https://www.ncbi.nlm.nih.gov/nucleotide/XM_037550413.1?report=genbank&log$=nucltop&blast_rank=10&RID=842NJS9E015) |
|  |  |  |  | PREDICTED: Hordeum vulgare subsp. vulgare WD repeat-containing protein 91 homolog (LOC123430797), transcript variant X2, mRNA | 88% | 0 | 94.63 | [XM_045114644.1](https://www.ncbi.nlm.nih.gov/nucleotide/XM_045114644.1?report=genbank&log$=nucltop&blast_rank=11&RID=842NJS9E015) |
|  |  |  |  | PREDICTED: Hordeum vulgare subsp. vulgare WD repeat-containing protein 91 homolog (LOC123430797), transcript variant X3, misc_RNA | 74% | 0 | 95.59 | [XR_006622986.1](https://www.ncbi.nlm.nih.gov/nucleotide/XR_006622986.1?report=genbank&log$=nucltop&blast_rank=12&RID=842NJS9E015) |
|  |  |  |  | PREDICTED: Triticum aestivum WD repeat-containing protein 91 homolog (LOC123181015), transcript variant X2, misc_RNA | 98% | 0 | 96.51 | [XR_006491443.1](https://www.ncbi.nlm.nih.gov/nucleotide/XR_006491443.1?report=genbank&log$=nucltop&blast_rank=13&RID=842NJS9E015) |
| IWB11406 | TraesCS1B03G1027100 | LOC123142563 | XM_044561428.1 | Triticum aestivum mRNA, clone: tplb0014f10, cultivar Chinese Spring | 100% | 0 | 98.32 | [AK456918.1](https://www.ncbi.nlm.nih.gov/nucleotide/AK456918.1?report=genbank&log$=nucltop&blast_rank=2&RID=8421BR0W015) |
|  |  |  |  | PREDICTED: Triticum dicoccoides nucleosome assembly protein 1;2-like (LOC119349225), mRNA | 100% | 0 | 96.73 | [XM_037617257.1](https://www.ncbi.nlm.nih.gov/nucleotide/XM_037617257.1?report=genbank&log$=nucltop&blast_rank=3&RID=8421BR0W015) |
|  |  |  |  | PREDICTED: Triticum aestivum nucleosome assembly protein 1;2 (LOC123065052), transcript variant X2, mRNA | 100% | 0 | 95.52 | [XM_044488428.1](https://www.ncbi.nlm.nih.gov/nucleotide/XM_044488428.1?report=genbank&log$=nucltop&blast_rank=4&RID=8421BR0W015) |
|  |  |  |  | PREDICTED: Triticum dicoccoides nucleosome assembly protein 1;2 (LOC119288874), transcript variant X2, mRNA | 99% | 0 | 95.48 | [XM_037568390.1](https://www.ncbi.nlm.nih.gov/nucleotide/XM_037568390.1?report=genbank&log$=nucltop&blast_rank=5&RID=8421BR0W015) |
|  |  |  |  | PREDICTED: Triticum urartu nucleosome assembly protein 1;2 (LOC125526742), mRNA | 100% | 0 | 94.94 | [XM_048691382.1](https://www.ncbi.nlm.nih.gov/nucleotide/XM_048691382.1?report=genbank&log$=nucltop&blast_rank=6&RID=8421BR0W015) |
|  |  |  |  | PREDICTED: Triticum aestivum nucleosome assembly protein 1;2 (LOC123182812), mRNA | 99% | 0 | 92.68 | [XM_044595482.1](https://www.ncbi.nlm.nih.gov/nucleotide/XM_044595482.1?report=genbank&log$=nucltop&blast_rank=7&RID=8421BR0W015) |
|  |  |  |  | PREDICTED: Aegilops tauschii subsp. strangulata nucleosome assembly protein 1;2 (LOC109745667), mRNA | 100% | 0 | 92.06 | [XM_020304783.4](https://www.ncbi.nlm.nih.gov/nucleotide/XM_020304783.4?report=genbank&log$=nucltop&blast_rank=8&RID=8421BR0W015) |
|  |  |  |  | PREDICTED: Triticum aestivum nucleosome assembly protein 1;2 (LOC123065052), transcript variant X1, mRNA | 99% | 0 | 94.82 | [XM_044488421.1](https://www.ncbi.nlm.nih.gov/nucleotide/XM_044488421.1?report=genbank&log$=nucltop&blast_rank=9&RID=8421BR0W015) |
|  |  |  |  | PREDICTED: Triticum dicoccoides nucleosome assembly protein 1;2 (LOC119288874), transcript variant X1, mRNA | 99% | 0 | 94.82 | [XM_037568389.1](https://www.ncbi.nlm.nih.gov/nucleotide/XM_037568389.1?report=genbank&log$=nucltop&blast_rank=10&RID=8421BR0W015) |
|  |  |  |  | Hordeum vulgare subsp. vulgare mRNA for predicted protein, complete cds, clone: NIASHv2064P18 | 100% | 0 | 90.52 | [AK367925.1](https://www.ncbi.nlm.nih.gov/nucleotide/AK367925.1?report=genbank&log$=nucltop&blast_rank=11&RID=8421BR0W015) |
|  |  |  |  | PREDICTED: Hordeum vulgare subsp. vulgare nucleosome assembly protein 1;2 (LOC123448266), mRNA | 100% | 0 | 90.52 | [XM_045125101.1](https://www.ncbi.nlm.nih.gov/nucleotide/XM_045125101.1?report=genbank&log$=nucltop&blast_rank=12&RID=8421BR0W015) |
